# Post-Acute Care Interventions in Patients Hospitalized Due to COPD Exacerbation Before and After Implementation of an Integrated Care Program

**DOI:** 10.2147/COPD.S496167

**Published:** 2025-01-28

**Authors:** Christine Hübsch, Christian F Clarenbach, Daniel P Franzen, Gabriela Schmid-Mohler

**Affiliations:** 1 Centre of Clinical Nursing Science, University Hospital Zurich, Zurich, Switzerland; 2 Division of Pulmonology, University Hospital Zurich, Zurich, Switzerland; 3 Faculty of Medicine, University of Zurich, Zurich, Switzerland

**Keywords:** COPD, exacerbation, implementation, post-acute care, nursing

## Abstract

**Purpose:**

In Switzerland, while the quality of acute inpatient care for patients with AECOPD is high, a lack of post-acute care interventions has been identified. To correct this shortfall, an integrated care program for patients with AECOPD was initiated at University Hospital Zurich. The study’s aim was to compare defined post-acute care intervention implementation rates before and after the new program’s implementation.

**Methods:**

A retrospective medical chart review was performed regarding patients hospitalized due to AECOPD between July 2019 and March 2023. The control group (CG) had received usual care, while the intervention group (IG) received the newly implemented program. Implementation rates were compared with Pearson’s chi-squared-test or Fisher’s exact test.

**Results:**

Charts of 107 participants (IG: 55, CG: 52) were evaluated. Implementation rates increased significantly in the IG for exacerbation management, dyspnea management, recommendation for rehabilitation, smoking cessation advice, evaluation of inhalation technique and recommendation of vaccination (p < 0.05) but not for physical activity, post-discharge medical follow-up or nutrition.

**Conclusion:**

This study provides promising evidence that the introduction of a hospital-initiated integrated care program can significantly increase the implementation rate of post-acute care interventions in patients hospitalized due to AECOPD.

## Introduction

In Switzerland, approximately 400’000 persons live with COPD.^1^ This condition’s most common complication is acute exacerbation of COPD (AECOPD), which is associated with disease progression and increased hospitalizations, leading to higher illness-related costs and excess mortality.^2^,^3^ Beneficial health behaviors, eg, early exacerbation recognition, symptom management, smoking cessation, regular physical activity, healthy diet, medication adherence, pneumococcal and influenza vaccination and dyspnea management, are known to reduce exacerbation and hospitalization rates.^3–5^ Structured and personalized self-management interventions addressing these behaviors are recommended for integration into standard inpatient and post-acute care.^3^,^6–9^ However, interventions that influence health behaviors are inadequately implemented in the hospital setting.^10^,^11^ Also, while the quality of acute inpatient care for patients with AECOPD is high in Switzerland, underuse of post-acute care interventions and coordinated self-management support across settings has been identified.^12^,^13^ In 2012 and 2013, only 1.9% of 263 hospitalized patients in three public hospitals in the Canton of Zurich received patient education and self-management support while hospitalized due to AECOPD.^12^ Barriers to the implementation of such interventions include the prioritization of acute care interventions, limited human resources, missing expertise in chronic care and behavior change and the lack of financial reimbursement.^13^,^14^ Moreover, the proportion of referrals for pulmonary rehabilitation in Switzerland is below 50%,^15^ despite evidence indicating that the timely initiation of pulmonary rehabilitation is beneficial in patients with AECOPD.^16^

At University Hospital Zurich, an integrated care program coordinated by a newly implemented advanced nursing practice (ANP) team was chosen to close this gap: the Nurse-led Integrated Care COPD (NICCO) program.^13^ This structured, patient-tailored behavioral program is initiated during hospitalization and continues for 13 weeks, mainly by telephone. The implementation of the NICCO program started in June 2020.^17^ To investigate the newly implemented program’s feasibility and effect, the NICCO pilot study was conducted using a monocentric parallel cluster design with a baseline period.^18^

The current sub-study of the NICCO study was founded on the assumption that adoption of the new integrated care program would increase the implementation rate of post-acute care interventions in patients hospitalized due to AECOPD. Therefore, its aim was to compare the implementation rates of defined post-acute care interventions before and after the integrated care program’s implementation.

## Materials and Methods

### The NICCO Program

The NICCO program’s development followed a Behavior Change Wheel (BCW)-based methodological approach to formulate and evaluate interventions concerning eight key elements of COPD management: 1. exacerbation and dyspnea management (using action plans); 2. physical activity; 3. referral to rehabilitation; 4. smoking cessation; 5. adherence to nutrition to prevent or manage underweight; 6. adherence to inhalation; 7. adherence to vaccination recommendations; and 8. post-discharge follow-up. The program also focuses on illness-related emotional distress and advanced end-of-life care planning.

While the ANP team—a team of nurses with doctoral, master’s, and bachelor’s degrees and/or experience in the field of pulmonology—leads the overall NICCO project, numerous other professionals (nurses, physicians, physiotherapists and, if needed, social workers, nutritionists and psychiatrists) also play important roles in the program. Beginning during patients’ inpatient stay for AECOPD, the program is delivered via face-to-face contact and lasts 13-weeks. During each patient’s stay, the ANP team, the responsible physician, a physiotherapist and the ward nurses deliver the various intervention packages that constitute the integrated care program (Table 1). For outpatient follow-up, ANP team members contact the patient—mainly by telephone—every one or two weeks for at least twelve weeks. Each contact includes a re-assessment of the patient’s target behaviors and individual progress within the program. A detailed description of the intervention is available elsewhere.^17^

**Table 1:** Overview of Post-Acute Care Interventions During Hospital Stay: Usual Care versus the Nurse-Led Integrated Care COPD (NICCO) Program

Post-Acute care Intervention and Evidence	Usual Care		NICCO Program	
	Content	Responsible Profession	Content	Responsible Profession
Exacerbation Management:Self-management interventions with exacerbation action plan improve health-related quality of life and reduce readmission risk^3^,^8^,^19–21^	No standardized procedure	Physician	Assessment and intervention regarding symptom management. Decision, recommendation and instruction of action plan	ANP Team
			Prescription and explanation of the medications in the action plan	Physician
Dyspnea Management:Relieving dyspnea limits disability, reduces distress, improves quality of life and reduces doctor visits^3^	Situation-specific assessment and intervention regarding dyspnea management	Ward Nurse	Reduce anxiety and dyspnea General assessment and intervention regarding dyspnea management	ANP Team
Physical Activity:Low physical activity is associated with higher mortality, increased risk for exacerbations and hospitalisations^25^,^26^	Prescription of physiotherapy in selected patients	Physician	Exercising 30 minutes per day and supervised exercise Information about the importance of exercise. Prescription of physiotherapy in all patients	Physician
	Assessment and intervention if there is a prescription	Physiotherapist	Assessment and intervention in all patients. Preparation of a movement plan	Physiotherapist
Rehabilitation:Pulmonary rehabilitation leads to lower mortality and fewer readmissions^16^,^27^	Discussion and prescription if required	Physician	Discussion and prescription if required	Physician
			Discuss expectations and goals regarding rehabilitation with the patient. Make a recommendation.	ANP Team
				Physiotherapist
Smoking Cessation:Quitting smoking and staying smoke-free are known to have the greatest capacity to influence prognosis and progression of COPD^3^	Information about the importance of smoking cessation. Prescription of nicotine replacement products	Physician	Assessment and Intervention. Referral to smoking cessation counseling if desired	ANP Team
	Offer and administer nicotine replacement products	Ward Nurse	Information about the importance of smoking cessation. Prescription of nicotine replacement products	Physician
			Offer and administer nicotine replacement products	Ward Nurse
			Making an appointment for outpatient smoking counseling	Smoking Cessation Counseling
Nutrition:Low body weight and malnutrition are common in patients with COPD and are related to disease progression^3^	Prescription of nutritional counseling for selected patients	Physician	Screening (NRS; BMI), Intervention if NRS≥4	ANP Team
			Intervention if NRS≥4	Nutritionist
Inhalation Adherence:Prevalence of inhalation non-adherence is high (22–93%)^28^; and up to 90% of patients inhale incorrectly 29,30	Assessment and Intervention regarding adherence and technique	Ward Nurse	Assessment and Intervention regarding adherence and technique	Ward Nurse
			Assess adherence (ZATA)	ANP Team
Influenza and Pneumococcal Vaccination:Influenza and pneumococcal vaccination decrease the incidence of lower respiratory tract infection^3^	Check vaccination status and make recommendation if necessary	Physician	Check vaccination status and make recommendation if necessary	ANP Team
				Physician
Post-discharge medical Follow-up:Early medical follow-ups of less than four weeks are associated with fewer exacerbation-related readmissions^3^	Write exit report and organize post-discharge medical follow-up	Physician	Write exit report and organize post-discharge medical follow-up	Physician
			Report and Email family doctor information about integrated care program and initiated self-management interventions	ANP Team

**Abbreviations**: BMI, body mass index; NRS, nutrition risk screening.

### Data Collection

We performed a retrospective medical chart review of patients hospitalized at University Hospital Zurich, Switzerland, between 1 July 2019 and 31 March 2023 due to AECOPD. In this tertiary academic referral center, patients with AECOPD usually stay either in the Division of Internal Medicine or in the Division of Pulmonology. For baseline data, charts were reviewed from July 2019 to June 2020 (control group). For data recorded after implementation of the NICCO program, charts were reviewed from June 2020 to March 2023 (intervention group). The control group received usual care, while the intervention group received the newly implemented NICCO program (Table 1). Inclusion criteria were a confirmed diagnosis of COPD, hospitalization due to a COPD exacerbation treated with steroids and/or antibiotics,^31^ age 18 or older and hospitalization on an Internal Medicine or Pulmonology ward. Exclusion criteria were cognitive impairment and not speaking any of our eleven supported languages, ie, German, French, Italian, English, Spanish, Portuguese, Serbian, Tamil, Hindi, Turkish or Slovakian. In cases of subsequent hospitalization due to AECOPD, only the first episode treated during the study period was considered. The study team identified prospective participants via regular screening of all in-patients’ electronic medical records. Eligible patients were contacted by the study team and provided with a participant information package. Patients were included either if they gave informed consent to participate in the NICCO study, ie, they allowed the collection of data from their electronic patient records, or if they gave general consent for data extraction from their records.

Data were collected using an anonymized case report form based on that used by Markun et al.^12^ Our form included sociodemographic characteristics, disease-specific data and variables regarding post-acute care interventions. Assessed post-acute care interventions were as follows: 1. exacerbation management (assessment, drafting and instruction of an action plan); 2. dyspnea management (interventions regarding dyspnea and/or anxiety); 3. interventions regarding physical activity; 4. recommendations for referral to rehabilitation; 5. smoking cessation advice (to current smokers); 6. nutrition counselling; 7. evaluation of inhalation technique; 8. assessment and recommendation of influenza and/or pneumococcal vaccination; and 9. post-discharge medical follow-up recommendations by physician.

Entries made to electronic patient records during their AECOPD hospitalization were searched for the specific interventions allocated to them, including reports from physicians, nurses, physiotherapists, nutritionists, and social services. We checked whether post-acute care interventions had been carried out (documented) during their hospitalization. Neither the interventions’ quality nor their results were evaluated. Data were entered by two master’s program nurses and double-checked by a study nurse. The first author randomly tested the data, making any necessary decisions in cases of ambiguity or disagreement between the raters’ assessments.

### Data Analysis

Descriptive statistics were calculated, ie, counts, percentages, medians and interquartile ranges (the ranges of values between each dataset’s 75th and 25th percentiles). To check for a difference between group allocation (control vs intervention group) regarding implementation rates, the Pearson’s chi-square test (X^2^) was performed. If the expected cell frequencies were ≤5, Fisher’s exact test was used. Statistical analyses used the SPSS Statistics, version 29 software package.^32^ For the sociodemographic characteristics and disease-specific data, missing data were excluded from the median and interquartile range calculations. For the interventions, missing data (no documentation found) were counted as not performed.

### Ethical Considerations

This study was part of the “Nurse-led integrated care to improve quality of life in COPD patients with a pulmonary exacerbation (NICCO)” study (www.kofam.ch, SNCTP000003402) and approved by the governmental ethics committee (BASEC No. 2019–00797). The study followed the principles of the Declaration of Helsinki.^33^ After participants were informed in writing and orally about the study, and understood that participation was voluntary, all signed written consent for the retrieval of their data from their electronic patient records. Data anonymization and confidentiality were ensured.

## Results

### Participants

Data from both the control (n = 52) and the intervention group (n = 55) were included in the electronic chart review. The overall median length of stay was 8 days (IQR: 6–10; control group median: 7 days (IQR: 6–10); intervention group median: 8 days (IQR: 6–11)). For participants’ sociodemographic characteristics and disease-specific data, see Table 2.

**Table 2:** Sociodemographic Characteristics and Disease-Specific Participant Data

Variable		Control Group (n=52) n or Median (% or IQR)	Intervention Group (n=55) n or Median (% or IQR)	Overall (n=107) n or Median (% or IQR)
Sociodemographic Characteristics	Age (years)	68.5 (62–76)	69 (63–75)	69 (62–75)
	Gender (male)	29 (55.8%)	29 (52.7%)	58 (54.2%)
	BMI (kg/m^2^)	23.7 (21–28)	22.8 (19–28)	23.3 (20–28)
	Missing	0 (0%)	1 (1.8%)	1 (0.9%)
Social Situation and Support	Living alone	27 (51.9%)	24 (43.5%)	51 (47.7%)
	Missing	4 (7.7%)	0 (0%)	4 (3.7%)
	Paid support and/ or support from social environment	27 (51.9%)	35 (63.6%)	62 (58%)
	Missing	6 (11.5%)	1 (1.8%)	7 (6.5%)
GOLD Stage	1	1 (1.9%)	0 (0%)	1 (0.9%)
	2	13 (25%)	9 (16.4%)	22 (20.6%)
	3	18 (34.6%)	20 (36.4%)	38 (35.5%)
	4	14 (26.9%)	23 (41.8%)	37 (34.6%)
	Missing	6 (11.6%)	3 (5.4%)	9 (8.3%)
Smoking Status	Pack years	50 (35–82)	50 (39–81)	50 (39–81)
	Missing	3	2	5
	Never smoker	2 (3.8%)	0 (0%)	2 (1.9%)
	Ex-smoker	36 (69.2%)	38 (69.1%)	74 (69.2%)
	Current smoker	14 (26.9%)	17 (30.9%)	31 (29%)
Oxygen Therapy	Having long-term oxygen prescription	24 (46.2%)	25 (45.5%)	49 (45.8%)
	Missing	15 (28.8%)	7 (12.7%)	22 (20.6%)
Comorbidities	Diabetes mellitus	9 (17.3%)	10 (18.2%)	19 (17.8%)
	Missing	2 (3.8%)	2 (3.6%)	4 (3.7%)
	Hypertension	19 (36.5%)	21 (38.2%)	40 (37.4%)
	Ischemic heart disease	5 (9.6%)	2 (3.6%)	7 (6.5%)
	Coronary artery disease	14 (26.9%)	17 (30.9%)	31 (29%)
	Systolic or diastolic dysfunction of the left ventricle	1 (1.9%)	5 (9.1%)	6 (5.6%)
	Pulmonary hypertension	3 (5.8%)	4 (7.3%)	7 (6.5%)
	Depression or Anxiety disorder	4 (7.7%)	8 (14.5%)	12 (11.2%)
	Substance Abuse (drugs; medication; alcohol)	6 (11.5%)	5 (9.1%)	11 (10.3%)

**Abbreviations**: BMI, body mass index; IQR, interquartile range; n, number.

### Implementation of Post-Acute Care Interventions

**Figure 1:**
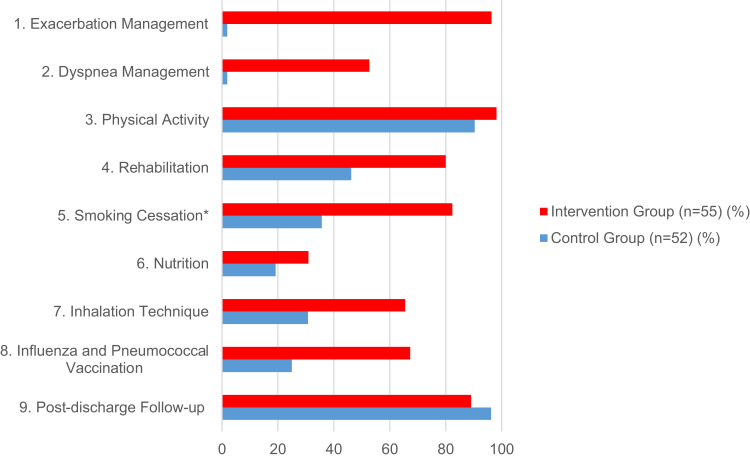
Implementation rates of control and intervention group.

**Notes**: *Smoking Cessation, subgroup of currently smoking patients (n = 14 (Control group); n = 17 (Intervention group)).

The implementation rates of almost all assessed post-acute care interventions were higher in the intervention group. Only the implementation rate of the intervention regarding post-discharge medical follow-up was higher in the control group. The highest implementation rate (98.2%) was for the physical activity intervention (in the intervention group); the two lowest were exacerbation and dyspnea management (both 1.9%) (in the control group). For interventions and their implementation rates in both groups, as well as the corresponding measure of association, see Table 3. For all measured implementation rates of both groups, see Figure 1.

**Table 3:** Interventions and Their Implementation Rates

Intervention	Interventions and Implementation rates n (%)			Measure of Association p-value; X^2^; Fisher’s Exact Test
	Control Group (n=52)	Intervention Group (n=55)	Total (n=107)	Link Between Group Allocation and Implementation Rate
1) Exacerbation Management	1 (1.9%)	53 (96.4%)	54 (50.5%)	p<0.001; X^2^(1)=95.367
2) Dyspnea Management	1 (1.9%)	29 (52.7%)	30 (28.0%)	p<0.001; X^2^(1)=34.193
3) Physical Activity	47 (90.4%)	54 (98.2%)	101 (94.4%)	Fisher’s Exact Test (two-tailed) p=0.106
4) Rehabilitation	24 (46.2%)	44 (80%)	68 (63.6%)	p<0.001; X^2^(1)=13.219
5) Smoking Cessation (in current smokers)	5 (35.7%) (n=14)	14 (82.4%) (n=17)	19 (61.3%) (n=31)	p=0.008; X^2^(1)=7.039
6) Nutrition	10 (19.2%)	17 (30.9%)	27 (25.2%)	p=0.148; X^2^(1)=2.094
7) Inhalation Technique	16 (30.8%)	36 (65.5%)	52 (48.6%)	p<0.001; X^2^(1)=14.495
8) Influenza and Pneumococcal Vaccination	13 (25%)	37 (67.3%)	50 (46.7%)	p<0.001; X^2^(1)=19.188
9) Post-discharge Follow-up	50 (96.2%)	49 (89.1%)	99 (92.5%)	Fisher’s Exact Test (two-tailed) p = 0.438

**Abbreviations**: n, number; X^2^, Pearson’s chi-square test.

## Discussion

In the current study, we compared defined post-acute care interventions before and after the implementation of a hospital-initiated integrated care program for patients with AECOPD. The implementation rates ranged from very low to very high, with significantly higher rates in the intervention group in six of the nine studied interventions (p < 0.05). Therefore, the assumption that the new integrated care program would lead to an increase in the implementation rate of post-acute care interventions in patients with AECOPD can be preliminarily confirmed.

Our results align with those of other studies,^12^,^34–36^ confirming both that post-acute care interventions are poorly implemented in the majority of institutions and that integrated care programs can improve implementation rates. However, the corroborating studies’ data sources, participants, interventions and settings are not directly comparable.

For example, in 2013, when Markun et al^12^ performed a retrospective medical chart review in three hospitals in Zurich, Switzerland, they found that implementation rates for seven post-acute care interventions were generally low. With implementation rates of 1.9% each, *patient education* and *self-management* were the least frequently applied. As our control group data from 2020 show, some implementation rates had not improved in at least seven years. While Markun et al’s^12^ data were drawn from documentation generated by the attending physicians, we analyzed the available documentation for all involved health professionals. This may have contributed to our intervention group’s significantly better results.

When Kaufmann et al^34^ conducted a retrospective medical chart review in an outpatient setting in Switzerland, the patients examined had no exacerbation and, on average, significantly less advanced COPD than ours. One private practice (medical records n = 20) that had implemented the “living well with COPD” patient self-management program^37^ showed significantly better documentation for nearly all measured interventions (p < 0.01) compared to the practices without this program. While such results attest to the value of that patient self-management program, differences between their patient sample and ours make it difficult to compare that practice’s results with ours. The majority of our sample’s patients had advanced COPD and were hospitalized. Further, their impaired physical condition may also have been related to a lower capacity and motivation to absorb and act on new knowledge regarding their COPD.

In an international European cluster randomized controlled trial,^36^ where patients with AECOPD received the usual care in the control group and a new COPD care pathway in the intervention group, process indicators were compared. The authors concluded that performance on key evidence-based interventions was better after implementation of the new care pathway. However, the setting and the care pathway in their study are not directly comparable to ours (eg, not Nurse-led).

Our intervention group showed a very clear intervention effect, with implementation rates higher—and in several cases, far higher—for eight of the nine studied interventions. Nevertheless, there is room for improvement regarding implementation rates. Possible reasons why implementation is not fully realized in most interventions are as follows: 1. Some interventions, eg, those regarding nutrition in patients with Nutrition Risk Screening (NRS) ≥4, are delivered post-discharge.^17^ 2. In others, eg, to promote smoking cessation, the healthcare staff may be reluctant to address the topic.^17^ 3. Continuity and regular training of healthcare professionals, eg, the nurses on the ward, cannot be guaranteed at all times.^14^ 4. As hospital stays are often short, delayed prescriptions, eg, for physiotherapy, have been identified as barriers to intervention implementation.^14^ 5. The ANP team has limited or no influence on post-discharge medical follow-up.

Overall, the intervention group’s implementation rates show a successful implementation of the NICCO program. This level of success may have been facilitated by a number of exceptional factors, eg, the allocation of additional personnel resources, the presence of an ANP team, and the integration of the NICCO intervention into a broader care program. This program is based on a trustful relationship between the patients and the ANP team, whose members also address COPD-linked psychosocial aspects and burdensome emotions.^17^

Based on this study’s results, the following can be recommended to further develop the NICCO intervention: 1. Monitor the nutritional screening rate in order to start nutritional supplementation during hospitalization if indicated.^22^ 2. Optimize evaluation and instruction of inhalation technique by having the ANP team take it over. 3. Include current vaccination recommendations for further development of the intervention, which now include vaccinations against SARS-CoV-2, zoster; diphtheria, tetanus, and pertussis (Tdap).^3^

## Limitations

This study’s most notable limitations spring from our use of a retrospective medical chart review as a design. Operationalization was challenging, and inter-study comparison is limited.^23^ Further, retrospective designs entail a risk of underreporting;^24^ and our group assignments were not randomized. However, to ensure consistency of measurement, two qualified persons independently rated the studied interventions, after which the first author reviewed their ratings and made final decisions in cases of disagreement or ambiguity.

## Conclusion

This study provides promising evidence that the introduction of a hospital-initiated integrated care program can significantly increase the implementation rate of selected post-acute care interventions in patients hospitalized due to COPD exacerbation.

## Data Availability

In light of the sensitive nature of personal data, it is not available for unrestricted access. Any queries relating to data sharing should be directed to the corresponding author.

## References

[1] Federal Office of Public Health (FOPH). Chronic Respiratory Diseases (German). Federal Office of Public Health (FOPH), Available from: https://www.bag.admin.ch/bag/de/home/krankheiten/krankheiten-im-ueberblick/chronische-atemwegserkrankungen.html. Accessed April 28, 2022.

[2] Gutierrez Villegas C, Paz-Zulueta M, Herrero-Montes M, Paras-Bravo P, Madrazo Perez M. Cost analysis of chronic obstructive pulmonary disease (COPD): a systematic review. *Health Econ Rev*. 2021;11(1):31. doi:10.1186/s13561-021-00329-9 34403023 PMC8369716

[3] Global Initiative for Chronic Obstructive Lung Disease. Global strategy for the diagnosis, management, and prevention of COPD: 2023 report. 2023. Available from: https://goldcopd.org/2023-gold-report-2/. Accessed January 01, 2025.

[4] Moy ML, Teylan M, Weston NA, Gagnon DR, Garshick E. Daily step count predicts acute exacerbations in a US cohort with COPD. *PLoS One*. 2013;8(4):e60400. doi:10.1371/journal.pone.0060400 23593211 PMC3617234

[5] Poole PJ, Chacko E, Wood-Baker RW, Cates CJ. Influenza vaccine for patients with chronic obstructive pulmonary disease. *Cochrane Database Syst Rev*. 2006;(1):Cd002733. doi:10.1002/14651858.CD002733.pub2 16437444

[6] Howcroft M, Walters EH, Wood-Baker R, Walters JA. Action plans with brief patient education for exacerbations in chronic obstructive pulmonary disease. *Cochrane Database Syst Rev*. 2016;12:CD005074. doi:10.1002/14651858.CD005074.pub4 27990628 PMC6463844

[7] Poot CC, Meijer E, Kruis AL, Smidt N, Chavannes NH, Honkoop PJ. Integrated disease management interventions for patients with chronic obstructive pulmonary disease. *Cochrane Database Syst Rev*. 2021;9:CD009437. doi:10.1002/14651858.CD009437.pub3 34495549 PMC8425271

[8] Zwerink M, Brusse-Keizer M, van der Valk PD, et al. Self management for patients with chronic obstructive pulmonary disease. *Cochrane Database Syst Rev*. 2014;2014(3):Cd002990. doi:10.1002/14651858.CD002990.pub3 24665053 PMC7004246

[9] Long H, Howells K, Peters S, Blakemore A. Does health coaching improve health-related quality of life and reduce hospital admissions in people with chronic obstructive pulmonary disease? A systematic review and meta-analysis. *Br J Health Psychol*. 2019;24(3):515–546. doi:10.1111/bjhp.12366 31033121 PMC6767143

[10] Calle Rubio M, Lopez-Campos JL, Soler-Cataluna JJ, et al. Variability in adherence to clinical practice guidelines and recommendations in COPD outpatients: a multi-level, cross-sectional analysis of the EPOCONSUL study. *Respir Res*. 2017;18(1):200. doi:10.1186/s12931-017-0685-8 29197415 PMC5712134

[11] Seys D, Bruyneel L, Decramer M, et al. An international study of adherence to guidelines for patients hospitalised with a COPD exacerbation. *COPD*. 2017;14(2):156–163. doi:10.1080/15412555.2016.1257599 27997254

[12] Markun S, Franzen DP, Dalla Lana K, et al. Acute exacerbated COPD: room for improvement in key elements of care. *Int J Chron Obstruct Pulmon Dis*. 2017;12:2969–2975. doi:10.2147/copd.S145496 29066878 PMC5644547

[13] Schmid-Mohler G, Clarenbach C, Brenner G, et al. Advanced nursing practice in COPD exacerbations: the solution for a gap in Switzerland? *ERJ Open Res*. 2020;6(2):00354–2019. doi:10.1183/23120541.00354-2019 32577416 PMC7293988

[14] Hübsch C, Clarenbach C, Chadwick P, et al. Acceptability, appropriateness and feasibility of a nurse-led integrated care intervention for patients with severe exacerbation of COPD from the healthcare professional’s perspective - A mixed method study. *Int J Chron Obstruct Pulmon Dis*. 2023;18:1487–1497. doi:10.2147/copd.S404712 37489242 PMC10363352

[15] Buess M, Schilter D, Schneider T, et al. Treatment of COPD exacerbation in Switzerland: results and recommendations of the European COPD audit. *Respiration; International Review of Thoracic Diseases*. 2017;94(4):355–365. doi:10.1159/000477911 28719893

[16] Ryrsø CK, Godtfredsen NS, Kofod LM, et al. Lower mortality after early supervised pulmonary rehabilitation following COPD-exacerbations: a systematic review and meta-analysis. *BMC Pulm Med*. 2018;18(1):154. doi:10.1186/s12890-018-0718-1 30219047 PMC6139159

[17] Schmid-Mohler G, Hübsch C, Steurer-Stey C, et al. Supporting behavior change after AECOPD - development of a hospital-initiated intervention using the behavior change wheel. *Int J Chron Obstruct Pulmon Dis*. 2022;17:1651–1669. doi:10.2147/COPD.S358426 35923357 PMC9339665

[18] Schmid-Mohler G, Hübsch C, Braun J, et al. Effect of a nurse-led integrated care intervention on quality of life and rehospitalisation in Patients with severe exacerbation of COPD – a pilot study. *Chron Respir Dis*. 2024 21: doi:10.1177/14799731241291067. PMC11481074 39407408

[19] National Institute for Health and Care Excellence NICE. National Institute for Health and Care Excellence: Guidelines. Chronic obstructive pulmonary disease in over 16s: diagnosis and management. London: National Institute for Health and Care Excellence (NICE); 2019. 31211541

[20] Lenferink A, Brusse-Keizer M, van der Valk P DLPM, Frith P A, Zwerink M, Monninkhof E M, van der Palen J, Effing T W and. (2017). Self-management interventions including action plans for exacerbations versus usual care in patients with chronic obstructive pulmonary disease. *Cochrane Database Syst Rev.*, 2017(8), doi:10.1002/14651858.CD011682.pub2 PMC6483374 28777450

[21] Benzo R P, Abascal-Bolado B and Dulohery M M. (2016). Self-management and quality of life in chronic obstructive pulmonary disease (COPD): The mediating effects of positive affect. *Patient Educ Couns*, 99(4), 617–623. doi:10.1016/j.pec.2015.10.031 26632024 PMC4808334

[22] Ferreira IM, Brooks D, White J, Goldstein R. Nutritional supplementation for stable chronic obstructive pulmonary disease. *Cochrane Database Syst Rev*. 2012;(12). doi:10.1002/14651858.CD000998.pub3 PMC11742366 23235577

[23] Vassar M, Holzmann M. The retrospective chart review: important methodological considerations. *J Educ Eval Health Prof*. 2013;10:12. doi:10.3352/jeehp.2013.10.12 24324853 PMC3853868

[24] Hess DR. Retrospective studies and chart reviews. *Respir Care*. 2004;49(10):1171–1174. 15447798

[25] Gimeno-Santos E *et al*. (2014). Determinants and outcomes of physical activity in patients with COPD: a systematic review. *Thorax*, 69(8), 731–739. doi:10.1136/thoraxjnl-2013-204763 24558112 PMC4112490

[26] Watz H *et al*. (2014). An official European Respiratory Society statement on physical activity in COPD. *Eur Respir J*, 44(6), 1521–1537. doi:10.1183/09031936.00046814 25359358

[27] Lindenauer P K, Stefan M S, Pekow P S, Mazor K M, Priya A, Spitzer K A, Lagu T C, Pack Q R, Pinto-Plata V M and ZuWallack R. (2020). Association Between Initiation of Pulmonary Rehabilitation After Hospitalization for COPD and 1-Year Survival Among Medicare Beneficiaries. *JAMA*, 323(18), 1813–1823 doi:10.1001/jama.2020.4437 32396181 PMC7218499

[28] Bhattarai B, Walpola R, Mey A, Anoopkumar-Dukie S and Khan S. (2020). Barriers and Strategies for Improving Medication Adherence Among People Living With COPD: A Systematic Review. *Respir Care*, 65(11), 1738–1750. doi:10.4187/respcare.07355 32576706

[29] Souza M Luiza, Meneghini A Cristina, Ferraz É, Vianna E Oliveira and Borges M Carvalho. (2009). Knowledge of and technique for using inhalation devices among asthma patients and COPD patients. *J Bras Pneumol*, 35(9), 824–831. doi:10.1590/S1806-37132009000900002 19820807

[30] Sanchis J, Gich I and Pedersen S. (2016). Systematic Review of Errors in Inhaler Use. *CHEST*, 150(2), 394–406. doi:10.1016/j.chest.2016.03.041 27060726

[31] Global Initiative for Chronic Obstructive Lung Disease. Global strategy for the diagnosis, management, and prevention of COPD: 2022 report. 2021.

[32] IBM SPSS statistics for windows, version 29.0. *IBM Corp*. 2022.

[33] World Medical Association. Declaration of Helsinki: ethical principles for medical research involving human subjects. *JAMA*. 2013;310(20):2191–2194. doi:10.1001/jama.2013.281053 24141714

[34] Kaufmann AC, Markun S, Hasler S, et al. Performance measures in the management of chronic obstructive pulmonary disease in primary care: a retrospective analysis. *Praxis*. 2015;104(17):897–907. doi:10.1024/1661-8157/a002101 26286494

[35] Steurer-Stey C, Dallalana K, Jungi M, Rosemann T. Management of chronic obstructive pulmonary disease in Swiss primary care: room for improvement. *Qual Primary Care*. 2012;20(5):365–373. 23114004

[36] Vanhaecht K, Lodewijckx C, Sermeus W, et al. Impact of a care pathway for COPD on adherence to guidelines and hospital readmission: a cluster randomized trial. *Int J Chron Obstruct Pulmon Dis*. 2016;11:2897–2908. doi:10.2147/COPD.S119849 27920516 PMC5126002

[37] Living well with COPD. Living well with COPD. Available from: www.livingwellwithcopd.com. Accessed 15th, March, 2022.

